# Role of Inositol Poly-Phosphatases and Their Targets in T Cell Biology

**DOI:** 10.3389/fimmu.2013.00288

**Published:** 2013-09-23

**Authors:** Neetu Srivastava, Raki Sudan, William Garrow Kerr

**Affiliations:** ^1^Department of Microbiology and Immunology, SUNY Upstate Medical University, Syracuse, NY, USA; ^2^Department of Pediatrics, SUNY Upstate Medical University, Syracuse, NY, USA; ^3^Department of Biochemistry and Molecular Biology, SUNY Upstate Medical University, Syracuse, NY, USA; ^4^Department of Chemistry, Syracuse University, Syracuse, NY, USA

**Keywords:** SHIP1, SHIP2, T cells, T lymphocytes, adoptive T cell transfer, INPP4, PTEN, PI3K

## Abstract

T lymphocytes play a critical role in host defense in all anatomical sites including mucosal surfaces. This not only includes the effector arm of the immune system, but also regulation of immune responses in order to prevent autoimmunity. Genetic targeting of PI3K isoforms suggests that generation of PI(3,4,5)P_3_ by PI3K plays a critical role in promoting effector T cell responses. Consequently, the 5′- and 3′-inositol poly-phosphatases SHIP1, SHIP2, and phosphatase and tensin homolog capable of targeting PI(3,4,5)P_3_ are potential genetic determinants of T cell effector functions *in vivo*. In addition, the 5′-inositol poly-phosphatases SHIP1 and 2 can shunt PI(3,4,5)P_3_ to the rare but potent signaling phosphoinositide species PI(3,4)P_2_ and thus these SHIP1/2, and the INPP4A/B enzymes that deplete PI(3,4)P_2_ may have precise roles in T cell biology to amplify or inhibit effectors of PI3K signaling that are selectively recruited to and activated by PI(3,4)P_2_. Here we summarize recent genetic and chemical evidence that indicates the inositol poly-phosphatases have important roles in both the effector and regulatory functions of the T cell compartment. In addition, we will discuss future genetic studies that might be undertaken to further elaborate the role of these enzymes in T cell biology as well as potential pharmaceutical manipulation of these enzymes for therapeutic purposes in disease settings where T cell function is a key *in vivo* target.

## Introduction

**Figure 1:**
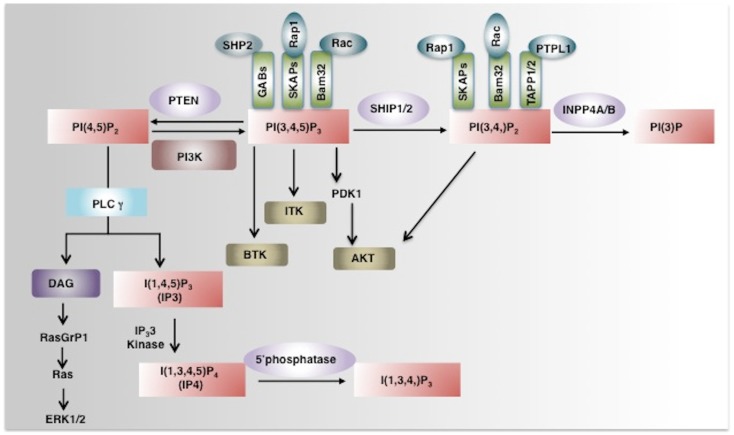
**Phosphoinositide signaling and its regulation by phosphatases**. PI3K converts PI(4,5)P_2_ to a key secondary messenger PI(3,4,5)P_3._ Phosphatases like PTEN and SHIP1/2 regulate cellular levels of PI(3,4,5)P_3_ by hydrolyzing it to PI(4,5)P_2_ and PI (3,4)P_2_ respectively. PLCγ converts PI(4,5)P_2_ to IP_3_ and DAG. IP_3_ a soluble inositol phosphate is required for Ca^2+^ mobilization while DAG can activate the Ras-Raf-ERK1/2 pathway. IP_3_ 3-Kinases convert IP_3_ to IP_4_, another important soluble inositol poly-phosphate that either positively or negatively regulates the binding of PI(3,4,5)P_3_ to PH-domain containing proteins. The SHIP1/2 product PI(3,4)P2 is hydrolyzed by INPP4A/B into PI(3)P by removal of the phosphate at the 4-position of the inositol ring. PI(3,4,5)P_3_ and/or PI(3,4)P_2_ enable recruitment to the plasma membrane of several PH-domain containing proteins including PDK1, AKT, BTK, ITK, and thus regulate pivotal cellular processes including activation, proliferation, and survival. PH-domain containing adaptor proteins (GABs, SKAPs, Bam32, and TAPP) can also bind to phosphoinositides and regulate cell signaling (indicated as green boxes). AKT, Protein Kinase B; PDK1, phosphoinositide-dependent kinase-1, PLCγ phospholipase Cγ; ITK, IL-2-inducible T cell kinase) and BTK, Bruton agammaglobulinemia tyrosine kinase.

Inositol phospholipid signaling pathway plays an integral role in development, proliferation, differentiation, and survival of lymphocytes (1–4). The principal second messenger of the PI3K pathway PtdIns(3,4,5)P_3_ is generated by phosphorylation of the 3′-hydroxyl group of PtdIns(4,5)P_2_ by PI3Ks. PI3Ks are grouped into three categories, Class I, II, and III on the basis of substrate specificity and structure. Only class I PI3Ks can use PtdIns(4,5)P_2_ to generate PtdIns(3,4,5)P_3_ at the inner leaflet of plasma membrane (5). PtdIns(3,4,5)P_3_ acts as binding site for several intracellular signaling molecules that containing a Pleckstrin-homology domain (PH-domain) and thus facilitates their recruitment to the plasma membrane. AKT/PKB is the most important PH-domain containing kinase required for cell growth, survival, and proliferation in most cell types and appropriately its PH-domain can bind PtdIns(3,4,5)P_3_ (6, 7). In addition to AKT, the PH-domain containing Tec family tyrosine kinases ITK (IL-2-inducible T cell kinase) and BTK (Bruton agammaglobulinemia tyrosine kinase) also have specificity for PtdIns(3,4,5)P_3_ and are important mediators of PI3K signaling pathway in T and B cells, respectively (Figure 1). Based on genetic models both class IA (p110α, p110β, and p110δ) and class IB PI3K (p110γ) play roles in thymocyte development. p110γ-knockout mice have increased apoptosis of DP thymocytes and double knockout p110δ/γ mice have significantly reduced number of thymocytes, a profound T cell lymphopenia and multiple organ inflammation (8–12). In addition to that mice with a knock-in point mutation of p110δ (p110deltaD910A/D910A) have severe defects in T cell receptor signaling and impaired Treg cell function (13–15). The T cell specific class IA PI3K deficient mice do not have defects in thymocyte and in peripheral T cell development, but they do exhibit defective TCR signaling, *in vitro* proliferation and cytokine production (16, 17). Altogether these findings demonstrate that the PI3K signaling pathway responsible for generation of PtdIns(3,4,5)P_3_ plays an important role in T cell development and activation and suggest that inositol poly-phosphatases like phosphatase and tensin homolog (PTEN), SHIP1, SHIP2, and INPP4A/B may have an opposing, or in some cases, a facilitating role downstream of PI3K in T lymphocytes.

The cellular pool of inositol phospholipids is determined in part by inositol phosphatases that by dephosphorylation of PtdIns(3,4,5)P_3_, can regulate PI3K-mediated signaling pathway. Three important phosphatases, which dephosphorylate PtdIns(3,4,5)P_3_ are PTEN, SHIP1, and SHIP2. PTEN is 3′ poly-phosphatase that converts PtdIns(3,4,5)P_3_ to PtdIns(4,5)P_2_ while the SHIP family phosphatases, SHIP1 and SHIP2, are 5′ poly-phosphatases, which convert PtdIns(3,4,5)P_3_ to PtdIns(3,4,)P_2_ (18, 19). The importance of these phosphatases in immune cell signaling was revealed by the demonstration that SHIP1 deficiency leads to severe myeloproliferative disorder and impaired NK cell function while mice with a conditional deletion of PTEN have impaired T cell immune responses (20–22). The present review focuses on the role of these inositol phosphatases in T cell biology.

## SHIP1 in T Cell Biology

SHIP1 (Src homology 2-containing inositol phosphatase) is a 5′-inositol poly-phosphatase that removes the 5′ phosphate from PtdIns(3,4,5)P_3_ and Ins(1,3,4,5)P_4_, thereby regulating PI3K signaling pathway (23). SHIP1 is expressed in hematopoietic cells, mesenchymal stem cells, and osteoblasts (24) as a 145/150 kDa doublet. SHIP1 contains an N-terminal SH2 domain, a central phosphatase domain, a C-terminal NPXY motifs, a Serine residue that can be phosphorylated by PKA, proline rich sequences as well as domains adjacent to the phosphatase domain that can recognize either its substrate or its product (23). The SH2 domain mediates binding of SHIP1 to ITAM and ITIM motifs in receptor tails such as the CD3 chains that associate with the TCR (25, 26) or with various adaptor proteins (27, 28). By virtue of its enzymatic as well as its non-enzymatic functions, SHIP1 is implicated in various signaling pathways related to proliferation, apoptosis, cytokine signaling in lymphocytes and myeloid cells (23). Germline SHIP1^−/−^ mice although viable after weaning develop profound infiltration of myeloid cells in the lungs and severe inflammation in the terminal ileum of the gut resembling human Crohn’s disease (29) which leads to their early demise (20, 30). SHIP1^−/−^ mice have increased number of myeloid cells in most of tissues, but are lymphopenic (20) and have a profound deficit of T cells in the gut (29) indicating diverse functions for SHIP1 signaling in myeloid cells and T lymphocytes.

## SHIP1 in T Cell Signaling

First demonstration of involvement SHIP1 in T cells came from the observation that ligation of CD3 or CD28 on T cells results in SHIP1 tyrosine phosphorylation and membrane re-localization (31). SHIP1 is thought to be a component of a signaling complex that includes LAT (linker for activation of T cells), Grb2, Dok (downstream of tyrosine kinase) 1, and Dok2 that negatively regulate TCR signaling (32). SHIP1 functions as an adaptor that is required for tyrosine phosphorylation of Dok1 and Dok2 and thus enables Dok1/2 anchoring to LAT to negatively regulate the Zap-70 and AKT kinases thus attenuating TCR signaling (32). Consistent with the proposed negative regulation of TCR signaling, SHIP1 together with adaptor Dok1 and Dok2 has also been shown to be associated with the CD4-mediated inhibitory signaling (33). SHIP1 can also negatively regulate activation and membrane localization of Tec Kinase, which plays an essential role in PLCγ activation upon TCR stimulation (34, 35). However, despite these biochemical studies suggesting SHIP1 limits TCR signaling splenic T cells isolated from germline SHIP1^−^/^−^ mice have defective TCR signaling as shown by their poor proliferation in response to TCR stimulation. In addition, SHIP1^−/−^ T cells fail to induce IL-2 and IFNγ upon PMA/ionomycin stimulation although they have elevated levels of CD69 and CD25 and dramatically reduced expression CD62L and CD45RB expression (36). However a T cell-restrictive deletion of SHIP1 (CD4CreSHIP^ΔIPflox^) that deletes SHIP1 at double positive thymocyte stage does not exhibit the same T cell phenotype observed in the germline SHIP1^−^/^−^ mice (37). SHIP1 deleted T cells in these mice do not regulate TCR signal strength and no difference in the phosphorylation status of AKT, ERK, Zap-70, PLCγ, or calcium influx was observed between SHIP1^−^/^−^ and WT T cells. Also, in contrast to the poor proliferation of T cells from germline deficient mice, T cells from CD4CreSHIP^ΔIPflox^ mice proliferate normally in response to TCR stimulation. The authors argued that the observed phenotype of T cells in germline SHIP1^−^/^−^ mice is due to pleiotropic effect of dysregulated immune system as a consequence of SHIP1-deficient environment (37, 38). However, we and others have found that the in-frame deletion strategy utilized still allows substantial expression of a near full-length version of SHIP1 that only lacks the enzyme domain (39). Because of SHIP1’s ability to function in cell signaling by masking binding sites on receptor tails for other regulatory kinases and phosphatases (40, 41) confounds interpretation of results from SHIP^ΔIPflox^ strain difficult.

## SHIP1 in T Cell Development

SHIP1 alone does not affect T cell development as no deficiencies in the development of T cells in the thymus was observed in either germline SHIP1^−^/^−^ deficient mice or in CD4CreSHIP^ΔIPflox^mice (20, 36, 37). However, a double knockout of SHIP1 and adaptor protein Dok1 plays an important role in T cell development since mice with combined deficiency of SHIP1 and Dok1 have significantly reduced total thymocyte numbers, percentage of CD4^+^ CD8^+^ double positive T cells and increased CD4^−^CD8^−^ double negative T cells (36).

SHIP1 has been shown to be required for both CD4^+^ and CD8^+^ T cell survival homeostasis at mucosal sites (29, 42). SHIP1^−^/^−^ mice develop spontaneous intestinal inflammation, the disease is highly demarcated and confined to the terminal ileum, which resembles classical human Crohn’s disease (29). The disease is characterized by severe reduction in CD4^+^ and CD8^+^ T cells in the lamina propria of SHIP1^−/−^ mice suggesting that SHIP1 is required for effector T cell persistence in the small intestine. Because T cells play an important role in normal immune surveillance to both commensal microorganism and pathogens, in their absence SHIP1-deficient Neutrophils and other myeloid cells over-respond resulting in lethal inflammation in SHIP1^−/−^ mice (29). The mechanism of selective loss of T cells in mucosal tissues is currently under investigation. T cell-restrictive SHIP1-deficient CD4CreSHIP^ΔIPflox^ reported by Tarasenko et al. have apparently no defect in T cell activation or T cell numbers in periphery; however, mucosal T cells were not examined in their report (37). Interestingly T cells from CD4CreSHIP^ΔIPflox^ mice show biased toward Th1 skewing and have defective production of Th2 cytokines IL-4, IL-5, and IL-13. Consistent with this T cells from CD4CreSHIP^ΔIPflox^ mice respond poorly to *in vivo* challenge to *Schistosoma mansoni* eggs, which normally induce a Th2 response. These cells also express elevated levels of T-bet which has been shown to regulate CD8 T cell function. Consistent with that CD8 T cells from CD4CreSHIP^ΔIPflox^ mice that also delete SHIP1 in CD8 T cells were more efficient in a cytotoxicity assay as compared to WT controls (37).

## Regulation of Tregs and Th17 Cells by SHIP1

SHIP1 has been shown to limit expansion both myeloid and T lymphoid immune-regulatory cell (30, 36, 43– 46). Peripheral T cells from SHIP1^−/−^ mice have significantly increased numbers of CD4^+^CD25^+^FoxP3^+^ conventional regulatory T cells (36, 45). They exhibit significantly higher levels of CD103, GITR, OX40, and FcRγII/III, which is associated with their regulatory function (45). SHIP1^−/−^ regulatory T cells are equally suppressive both *in vitro* and *in vivo* when compared to SHIP1-competent T regulatory cells (45). In addition to conventional regulatory T cells, SHIP1 deficiency also promotes the accumulation of CD4^+^CD25^−^ iTreg cells that express FoxP3 in the periphery that have suppressive function (45). Although SHIP1 deficiency seems to promote regulatory T cell expansion, the inflammatory environment brought about by SHIP1-deficient myeloid cells may also play a role in Treg cell development. An elegant study by Collazo et al. demonstrated that SHIP1 regulates Treg cell development and iTreg formation in both a T cell intrinsic and extrinsic manner. Both T cell specific deletion of SHIP1 in LckCreSHIP^flox/flox^ or myeloid cell-specific deletion in LysCreSHIP^flox/flox^ mice increased the peripheral pool of CD4^+^CD25^+^FoxP3^+^ regulatory T cells and CD4^+^CD25^−^ iTreg cells expressing FoxP3 (44). These results indicate that SHIP1 exerts both T cell intrinsic and extrinsic control over peripheral Treg cell development and conversion in the periphery. In contrast to this, Tarasenko et al. in the CD4CreSHIP^ΔIPflox^ model reported that SHIP1 deletion had no effect on Treg cell development. However, the concern noted above regarding residual expression of a near full-length SHIP1 mutant in SHIP^ΔIPflox^ mice hampers interpretation of this negative finding. Locke et al. also reported T cell-intrinsic function of SHIP1 in iTreg development. They showed that the ability of SHIP1^−/−^ CD4^+^CD25^−^CD45RB^high^ T cells to develop into Foxp3^+^ cells *in vitro* in presence of TGFβ alone or in combination with retinoic acid (RA) was much higher compared to WT T cells (47). Interestingly, FoxP3 can enhance the expression of miR-155 by binding to an intron within the DNA sequence of the miR-155 precursor RNA suggesting that FoxP3 could potentially maintain Treg numbers by suppressing SHIP1 expression through induction of miR-155 (48– 50). Altogether the above studies suggest a potent role for SHIP1 in T cell-intrinsic control of native Treg development and iTreg formation in the periphery. In contrast to SHIP1’s function in limiting Treg numbers, it has been shown to required for Th17 development. SHIP1^−/−^ T cells fail to differentiate into Th17 cells and this deficiency was accompanied by reduced IL-6 mediated phosphorylation STAT3 (47). SHIP1^−/−^ T cells have high basal level of T-bet, a transcription factor known to negatively regulate Th17 differentiation and lower levels of RORγt mRNA, and thus it is likely that the altered T cell differentiation are regulated by SHIP1 via its control of these transcription factors at the molecular level. Table 1 summarizes the function SHIP1 in T cells in different genetic mouse models.

**Table 1:** **T cell phenotypes of inositol poly-phosphatase mutant mice**.

No.	KO	Gene deletion	T cell phenotype	Reference
1	SHIP^−/−^	Germline SHIP1 deletion	Poor proliferation of T cells	Helgason et al. (20 ), Kerr et al. (29 ), Kashiwada et al. (36 ), Collazo et al.(45 )
			Elevated levels of CD69, CD25 on T cells, and reduced levels of CD69L, CD45RB	
			Increased CD4^+^CD25^+^FoxP3^+^ Tregs	
			Increased CD4^+^CD25^−^FoxP3^+^ iTregs	
			Reduced CD4^+^ and CD8^+^ T cells in the gut	
2	SHIP^−/−^DOK1^−/−^	Germline SHIP1 and DOK1 deletion	Reduced thymocytes	Kashiwada et al. (36 )
			Reduced CD+CD8+ T cells in thymus	
			Reduced CD8^+^ T cells in the spleen	
			Altered CD4:CD8 ratio	
			Increased CD4^+^CD25^+^FoxP3^+^ Tregs	
3	CD4CreSHIP^f/f^	SHIP1 deletion in T cells	Normal T cell development	Tarasenko et al. (37 )
			No defect in T cell activation	
			Reduced levels of TH2 cytokines IL-4, IL-5, and IL-13	
			CD8^+^ T cells are more cytotoxic	
4	LckCreSHIP^f/f^	SHIP1 deletion in T cells	Increased CD4^+^CD25^+^FoxP3^+^ Tregs	Collazo et al. (44 )
			Increased CD4^+^CD25^−^FoxP3^+^ iTregs	
5	LysCreSHIP^f/f^	SHIP1 deletion in myeloid cells	Increased CD4^+^CD25^+^FoxP3^+^ Tregs	Collazo et al. (44 )
			Increased CD4^+^CD25^−^FoxP3^+^ iTregs	
6	PTEN^−/+^	Heterozygous deletion of PTEN	Increased proliferation of T cells	Di Cristofano et al. (51 )
			Reduced AICD	
7	LckCre-PTEN^flox/−^	PTEN deletion in T cells/heterozygous deletion of PTEN other tissues	CD4^+^ T cell lymphoma	Suzuki et al. (22)
			Defect in thymic negative selection	
			Increased TH1/TH2 cytokines	
			T cells are resistant to apoptosis	
8	LckCre-PTEN^f/f^	PTEN deletion in T cells	T cells are hyper-responsive to TCR stimulation	Hagenbeek et al.(52 ), Walsh et al.(53 )
			Refracted to anergy induction	
			Reduced expansion of Tregs	
			Increased TH2 cytokine	
9	OX40^cre^PTEN^flox^	PTEN deletion in mature CD4^+^T cells	T cells are hyper-proliferative	Soond et al. (54)
			Secrete more cytokine	
			T cells are super-helper with enhanced inflammatory antibacterial and anti-tumor responses	

## SHIP1 in T Cell Migration

PI3K-associated pathways have been implicated in regulation of chemokine signaling and migration of cell toward chemokine gradient (55, 56). In a polarized plasma membrane PI3K accumulates at the leading edge of the migratory cells leading to localized production of PI(3,4,5)P_3_ and thereby regulating cell migration. Because SHIP1 regulates levels of PI(3,4,5)P_3_ at PI3K signaling complexes it stands to reason that it may then regulate chemotaxis. Nishio et al. demonstrated that SHIP1-deficient neutrophils fail to polarize PI(3,4,5)P_3_ at the leading edge of migrating cells resulting in the inefficient migration of neutrophils and reduced polarity in response to chemoattractants (57). In T cells enhanced chemotaxis in response to stromal cell-derived factor-1 (SDF1) has been reported with SHIP1^−/−^ thymocytes and splenic CD4^+^ T cells (58). Consistent with this enforced overexpression of SHIP1 in Jurkat T cells abrogated CXCL12 mediated chemotaxis by this cell line (59). However it was also shown that the chemotaxis of SHIP1^−/−^ lymphocytes with other chemokines was comparable with that of WT lymphocytes indicating that SHIP1 involvement in regulating chemotaxis may be chemokine specific (58). More recently Harris et al. by using a lentivirally expressed SHIP1-specific shRNA in human CD4^+^ T cells showed that although the directional chemotaxis toward CXCL11 was unaffected, the overall basic motility and morphology of T cells was impaired in SHIP1 knockdown (KD) primary human T cells (60). SHIP1 KD T cells exhibited increased actin polymerization and loss of microvilli projection upon stimulation with CXCL11. Formation of microvilli involves phosphorylation of ezrin/radixin/moesin (ERM) proteins and once the cell is activated microvilli are frequently lost due to Rac-mediated dephosphorylation of ERM proteins (61). SHIP1 seems to negatively regulate Rac activation and/or ERM phosphorylation through a non-catalytic function as pretreatment with the PI3K inhibitor Ly294002 fails to rescue microvilli disassembly (60). However, a partial rescue in ERM phosphorylation by a Rac inhibitor in SHIP1 KD T cells indicates that Rac independent pathways are also involved. Additionally, the PH-domain containing adaptor protein Bam32, that can bind to both PI(3,4,5)P_3_ and PI(3,4)P_2,_ the SHIP1 substrate and product, respectively, is required for Rac1 activation and efficient BCR-induced cell adhesion (62). Thus, it is possible that both SHIP1 catalytic and non-catalytic functions are required for chemotaxis and cytoskeletal rearrangement; however, mechanistic studies in T cell conditional SHIP1 mutants are required to better define specific functions of SHIP1 in regulating these processes in an *in vivo* setting.

## SHIP1 in T Cell Apoptosis

The PI3K pathway is largely associated with cellular survival and proliferation as its product PI(3,4,5)P_3_ is known to activate molecules required for cell survival and proliferation. Because SHIP1 degrades PI(3,4,5)P_3_, it is primarily considered a negative regulator of PI3K-mediated cell survival. Indeed, SHIP1 plays a pro-apoptotic function in myeloid, erythroid, and in some instances B cells (63–66). However, it appears to play an opposite function in T cells. For instance SHIP1 limits Fas-induced apoptosis in human primary T cells *ex vivo* and a leukemic T cell line (67). Jurkat T cells, which do not express SHIP1 at normal levels are very sensitive to FasL mediated apoptosis; however, when SHIP1 is over-expressed in Jurkat T cells they become resistant to H_2_O_2_ and FasL mediated apoptosis (67, 68). It is also reported that SHIP1 attenuates FcγRIIB mediated apoptosis in B cells and that the failure to recruit SHIP1 to the receptor results in enhanced apoptosis (69, 70). Importantly SHIP1^−/−^ mice are lymphopenic, and have profound deficiency of both CD4^+^ and CD8^+^ T cells in the gut indicating that SHIP1 might be required for T cell survival (20, 29). A selective deficiency of effector T cells at these sites might result in recruitment of myeloid cells, which subsequently leads to the lethal mucosal inflammation in both the lungs and gut of SHIP1^−/−^ mice (23, 29). Interestingly reconstitution of sub-lethally irradiated SHIP1^−/−^ mice with SHIP1-competent T cell graft protects them from mucosal inflammation and prolongs their survival. Moreover, SHIP1 is required for persistence of mature T cells in the periphery and at mucosal surfaces as SHIP1^−/−^ T cells are impaired for survival when forced to compete with SHIP1^+/+^ T cells for representation in the peripheral T cell pool of either immunocompetent or SCID hosts. Our preliminary studies indicate that SHIP1 mediated protection of T cell death at mucosal surfaces involves Fas-FasL death receptor pathway (42). Unlike myeloid cells in which SHIP1 appears to promote cell death, T cells require SHIP1 for their survival and persistence. A growing body of evidence implicates PI(3,4)P_2_, the SHIP1 product, in cell survival as it can more efficiently recruit and activate Akt (71, 72) and protects cancer cells from apoptosis induced by SHIP1 selective (43) and pan-SHIP1/2 inhibitors (73). Consistent with this role of PI(3,4)P_2_ and SHIP1/2 in promoting cell survival, increased levels of PI(3,4)P_2_ in INPP4A and INPP4B mutant mice promote cell transformation and tumorigenicity (74, 75). SHIP1 and SHIP2 should not only be considered terminators of PI3K-mediated survival pathway, but paradoxically also facilitators of such survival signaling. With the growing evidence of its anti-apoptotic role in T cells, and in various cancer cells, it is important to understand when and how SHIP1 promotes pro-apoptotic vs. anti-apoptotic signaling. Here both cell types and the involved receptor(s) are likely critical determinants of this positive vs. negative role for SHIP1 and SHIP2 in apoptosis.

## SHIP1 and Phosphoinositide-Binding PH-Domain Containing Adaptor Proteins

SHIP1’s role as a positive regulator of PI3K signaling pathway can also be attributed to the ability of PI(3,4)P_2_, the SHIP1 product to mediate recruitment of PH-domain containing adaptor proteins including SKAP adaptors (SKAP55 and SKAP-hom), Bam32 (also known as DAPP1), TAPP1, and TAPP2 (76). These adaptor proteins have differential ability to bind phosphoinositides, PI(3,4,5)P_3_ vs. PI(3,4)P_2_ (Figure 1) and also exhibit differential expression across immune cell types. For instance SKAP55 expression is relatively more restricted to T cells while SKAP-hom is more widely expressed in immune cells. Although Bam32 is restricted to hematopoietic cells, it is more abundant in B cells and expressed in lower levels in T cells, dendritic cells, and macrophages. TAPP proteins are widely expressed in all the tissues; however TAPP2 is more abundant in immune cells (76). TAPP1 and TAPP2 stand out among the adaptor proteins as they can only bind to PI(3,4)P_2_ (27, 77), while SKAP adaptors and Bam32 can bind to both PI(3,4,5)P_3_ vs. PI(3,4)P_2_ with equal affinity (76). Mice deficient in SKAP55, which predominantly functions in T cells have impaired TCR induced adhesion to integrin ligands suggesting a role of SKAP in PI3K-mediated integrin activation in lymphocytes (78). Bam32 has been implicated in BCR signaling of B cells as Bam32^−/−^ mice have defects in various aspects of B cell activation. Bam32^−/−^ B cells have impaired BCR-induced proliferation and defective T-independent antibody responses (62). Bam32 has also shown to be required for germinal center progression and antibody affinity maturation (79). Bam32^−/−^ B cells are defective in cell spreading presumably due impaired cytoskeleton rearrangement (76). In T cells Bam32 is required for TCR mediated ERK activation (80, 81). Thus, SHIP1 through hydrolysis of PI(3,4,5)P3 to PI(3,4)P_2_ could differentially regulate the recruitment of SKAP and Bam32 adaptors and thereby impact T cell signaling. This question merits further study in SHIP mutant T cells and in the mice mutants for these adaptor proteins.

SHIP1 has been shown to enhance membrane recruitment of TAPP1 and TAPP2, the only adaptor proteins known to exclusively bind PI(3,4)P_2_ (82). Recently a knock-in mouse model that express normal endogenous level of mutant TAPP1 and TAPP2 which are incapable of binding to PI(3,4)P_3_ has been made to understand their physiological functions (83). Interestingly, the defects observed in the B cells of TAPP KI mice showed remarkable similarities with that of SHIP1^−/−^ mice (84). TAPP KI mice have elevated levels of serum immunoglobulin, autoantibody production, and they show a lupus-like phenotype. Importantly AKT phosphorylation was significantly increased upon BCR cross linking in B cells purified from these mice enhancing their proliferation (84). This indicates that in the absence of TAPP adaptor proteins, the PI(3,4)P_2_ is available to promote AKT recruitment and resulting in increased proliferation and survival, consistent with the proposed positive function of SHIP1 in survival and proliferation (23). The precise role(s) of TAPP as adaptor proteins in T cells is relatively uncharacterized; however, TAPP can bind to PTPL1 which, has been shown to inhibit cytokine-induced TH1/TH2 differentiation (85). Therefore TAPP may potentially play a role in cytokine signaling in T lymphocytes by promoting membrane localization or activity of PTPL1. Although much is known about Bam32 and TAPP’s function in B cells, it remains to determine whether there is a physiological function for the adapters proteins that involves T cell signaling.

## SHIP1 and Soluble Inositol Phosphate IP_4_

In addition to hydrolysis of PI(3,4,5)P_3_, SHIP1 can also dephosphorylate soluble inositol-1,3,4,5 tetrakisphosphate (IP_4_) *in vitro* (18, 86). IP_4_ is generated by phosphorylation of Ins(1,4,5)P_3_ (IP_3_) at its 3-position by IP_3_ 3-Kinases (IP_3_3K) (Figure 1). Mammals express four IP3Ks; ItpkA/B/C and IPMK (IP multikinase). Lymphocytes predominantly express two Iptks, IptkB, and Iptkc, while IptkC is expressed in many tissues, expression of IptkB is restricted to hematopoietic cells and brain (2, 87). IP4 is required for T cell development as *Itpkb*^−/−^ mice, are severely immunocompromised and lack mature T cells because of a block at the CD4^+^CD8^+^ DP stage due to impaired positive selection in the thymus (88, 89). Interestingly, PLCγ mediated DAG-induced ERK activation which is essential for positive selection is profoundly impaired in *Itpkb*^−/−^ mice (90). IP_4_ strongly resembles the phosphate headgroup of PI(3,4,5)P_3_ and therefore it can bind to PH-domain containing proteins that also bind to PI(3,4,5)P_3_ (e.g., ITK, AKT) and perhaps several others (2). In T cells IP4 functions as a second messenger and regulates Itk membrane recruitment and activation upon TCR stimulation and therefore it is essential for full activation of ITK and its effector PLCγ (90). At physiological concentrations of IP4 in TCR stimulated T cells it promotes ITK binding to PI(3,4,5)P_3_, whereas at high IP4 concentrations it competes with PI(3,4,5)P_3_ for PH-domain binding (2). Because of the essential role of IP4 in T cell development and function it would be intriguing to know whether IP4 turnover at in primary T cells is regulated by SHIP1 5′phosphatase activity. This might be investigated by determining the measuring IP4 levels in SHIP1-deficient T cells (vs. WT) to provide evidence of negative regulation of IP4 by SHIP1 *in vivo*. If this appears to be the case, then it would then be interesting to test whether the increased IP4 concentration in SHIP1^−/−^ T cells results in diminished PI(3,4,5)P_3_ binding of PH-domain signaling proteins recruited to PI(3,4,5)P_3_ (e.g., ItK or AKT) to regulate T cell function.

## SHIP2

A close homolog of SHIP1 is the ubiquitously expressed 150 kDa protein SHIP2. Unlike SHIP1, whose expression is confined to hematolymphoid cells, osteoblasts (24), and mesenchymal stem cells (91). SHIP2 is expressed broadly in both hematopoietic and non-hematopoietic tissues such as brain, skeletal muscle, heart, liver, and kidney (92, 93). SHIP2 hydrolyzes the 5′ phosphate of PI(3,4,5)P_3_ *in vitro* and *in vivo* and has also been shown to dephosphorylate PI(4,5)P_2_ *in vitro* (94, 95). Thus, it may not be restricted to hydrolysis of the 5′PO_4_ groups on 3′ PO_4_-containing poly-phosphates, PI(3,4,5)P3 and I(1,3,4,5)P4, like its close homolog SHIP1. SHIP2 is tyrosine phosphorylated upon stimulation with stem cell factor (SCF), interleukin-3 (IL-3), and granulocyte-macrophage colony-stimulating factor (GM-CSF), which results in its association with SHC (src homologous and collagen gene). Suggesting that SHIP2, similarly to SHIP1, is linked to downstream signaling events after activation of hematopoietic growth factor receptors. SHIP2 plays a major role in negatively regulating insulin signaling in non-immune cells (93). Bruyns et al. reported that both SHIP1 and SHIP2 are expressed in human T lymphocytes with only SHIP2 protein levels increased after long-term stimulation of the TCR (96). SHIP2 has also been shown to associate with the SH3 domain of Tec kinase and inhibit Tec-mediated TCR signaling (35). However, a physiological role for SHIP2 in T cell biology and function remains to be demonstrated and defined.

In addition to SHIP1 and SHIP2, eight other 5′ phosphatases have been reported; OCRL1 (oculocerebrorenal syndrome of Lowe), synaptojanin1, synaptojanin 2, proline rich inositol poly-phosphate 5-phosphatase (PIPP), 72-5ptase/Type IV/Inpp5e, SKIP, INPP5B, and 5-phosphatase1. With the exception of 5-phosphatase1 that hydrolyzes only the soluble inositol phosphates Ins(1,4,5)P_3_ and Ins(1,3,4,5)P_4_, other phosphatases can dephosphorylate 5-phosphorylated phosphoinositides including PtdIns(4,5)P_2_, PtdIns(3,4,5)P_3_, PtdIns(3,5)P_2_, and soluble inositol phosphates although with variable efficiency (95). Some of these phosphatases are implicated in human diseases, for instance OCRL1 mutations are associated with Lowe’s syndrome and Dent 2 disease (95, 97), SKIP and 72-5ptase/Type IV/Inpp5e are implicated in insulin signaling and glucose homeostasis while Synaptogenin1 in mice is required for neuronal function. Overlapping functions for some of the 5′phosphatases have been reported for example OCRL1^−^/^−^ mice do not develop Lowe’s disease since loss of OCRL1 was compensated by a highly homologous protein Inpp5b (98). Thus far no immune phenotype has been reported in any mouse mutant of these other 5′ inositol phosphatases. However, further studies are merited to rigorously exclude a specific role in T cell signaling.

## Phosphatase and Tensin Homolog (PTEN)

Phosphatase and tensin homolog deleted on chromosome 10 was originally identified as a tumor suppressor gene, which negatively regulates cell survival and proliferation and is mutated in several cancers (99, 100). PTEN germline mutations are associated with several hereditary disorders characterized by hamartomas and increased cancer risk such as Cowden syndrome, Bannayan–Riley–Ruvalcaba syndrome, Proteus syndrome, and Proteus-like syndrome, collectively classified as PTEN hamartoma tumor syndrome (PHTS) (101). PTEN predominantly acts as a 3′ lipid phosphatase to oppose PI3K signaling by dephosphorylating PI(3,4,5)P_3_, a product of PI3K, at its 3′ hydroxyl position to yield PI(4,5)P_2_ (102). Other than its 3′ lipid phosphatase activity, PTEN also possess protein phosphatase activity and has been reported to dephosphorylate focal adhesion kinase (FAK) by direct binding (103). Homozygous PTEN^−/−^ knockout mice die early during embryogenesis, precluding analysis of PTEN role in various adult tissues and organs in germline mutant mice. However a wide range of information has been collected from studies using mice heterozygous for PTEN or lacking PTEN in various tissues using Cre-loxP models (21). Table 1 summarizes the function PTEN in T cells in different genetic mouse models.

Phosphatase and tensin homolog heterozygous mice show high tumor incidence, impaired Fas mediated cell death, and develop autoimmune disorders. T cells from these mice show increased proliferation, reduced activation induced cell death suggesting an important role of PTEN in T cell survival and activation (51). Studies from mice lacking PTEN in T cells revealed an important role for PTEN in T cell development, function, and homeostasis (22). Mice lacking PTEN in T cells (LckCre-PTEN^flox/−^) die prematurely due to CD4^+^ T cell lymphomas and develop symptoms of autoimmunity like autoantibody production and hypergammaglobulinemia. These mice show defective lineage commitment, altered thymic selection, and impaired peripheral tolerance. T cells from these mice were hyper-proliferative, secreted increased levels of Th1/Th2 cytokines, and were autoreactive. Resistance to apoptosis, increased AKT and ERK phosphorylation, and increased Bcl-X_L_ expression were observed in these T cells suggesting a vital role PTEN in regulation of T cell survival and apoptosis signaling (22). Hagenbeek et al. further confirmed the role of PTEN in T cell survival and development by analyzing LckCre-PTEN^flox/flox^ mice. They showed that in the absence of PTEN there is a diminished requirement for both IL-7R and pre-TCR signaling in T cell development and proliferation (52). PTEN deficient CD4^+^ T cells show hyper-responsiveness to TCR stimulation without requirement for co-stimulation signals and are refractory to anergy induction. Moreover, PTEN^−/−^ T cells show increased AKT and GSK3β phosphorylation and enhanced IL-2 production upon TCR stimulation. This suggests that by negatively regulating TCR signaling, PTEN sets a threshold for T cell activation and imposes a requirement for co-stimulation and thus regulates T cell anergy (104). PTEN regulates the response of Tregs to IL-2 and plays a negative role in IL-2R signaling in Tregs, which normally do not expand in response to IL-2 alone. However, when Treg cells are deficient in PTEN they can proliferate upon IL-2 stimulation without the requirement for TCR stimulation (53). PTEN deficient CD4 T cells also produce more Th2 cytokines (IL-4, IL-10) in response to TCR stimulation alone or in combination with CD28 suggesting a role of PTEN in regulation Th2 cytokine production (105). Thus PTEN negatively regulates the TCR signaling and the induction of key cytokines. Thus, efficient and sustained TCR signaling and cytokine responses by T cells requires down-modulation of PTEN which occurs following TCR stimulation (106). Further Cbl-b has been shown to regulate down-modulation of PTEN in response to TCR/CD28 stimulation by inhibiting PTEN association with Nedd4, which targets PTEN K13 for K63-linked polyubiquitination suggesting that multiple pathways may regulate PTEN in the context to TCR/CD28 stimulation (107).

Studies from various knockout models showed clearly that PTEN plays an important role as a tumor and autoimmunity suppressor. However, the mechanistic insights into the relationship of these two functions of PTEN in T cells revealed that these two functions of PTEN are distinct, context dependent and are mediated in T cells at different developmental stages. By using mice with deletion of PTEN in T cells (CD4CrePten^fl/fl^), Liu et al. demonstrated that T cell lymphomas arise in the thymus whereas autoimmunity was mediated by mature peripheral T cells (108). Subsequently Soond et al. studied the role of PTEN in mature CD4 T_H_ cells by using OX40CrePten^flox^ mice. Contrary to models of thymocyte-specific PTEN deletion OX40CrePten^flox^ mice did not develop lymphomas and autoimmunity even at an advanced age suggesting that PTEN does not act as a tumor suppressor or repressor of autoimmunity in mature T cells. In fact, PTEN deficient CD4 T_H_ cells produced increased concentrations of cytokines and were hyper-proliferative. The authors postulated that enhanced cytokine production turned PTEN deficient T_H_ cells into “super-helpers” as enhanced inflammatory, antibacterial, and anti-tumor responses were observed in OX40^cre^Pten^flox^ mice (54). Thus contrary to the prevalent view PTEN does not essentially always function as a tumor suppressor or immune-suppressor and can also, like SHIP1, have varied functions depending on cell type, developmental stage of cell, and biological context. This was further confirmed by a recent study by Locke et al. by employing a model where PTEN is deleted in post-thymic T cells. They observed enhanced cytokine production, proliferation, and activation of post-thymic PTEN deleted T cells. As observed earlier, these effects were associated with increased AKT activity. However, CD28 independence and anergy resistance were not observed (109). Enhanced cytokine production, antibacterial, and anti-tumor responses of PTEN deficient T cells argue that therapeutic strategies targeting pharmacological inhibition of PTEN may prove attractive in immunotherapeutic strategies that require enhanced T effector function. Recently small molecule inhibitors of PTEN has been identified and used *in vivo* without causing prominent toxicity (110, 111). However, further studies are required to assess the role of these inhibitors on T cell effector and regulatory functions before considering their use in immunotherapeutic approaches.

## Inositol Poly-Phosphate 4-Phosphatase (INPP4)

**Figure 2:**
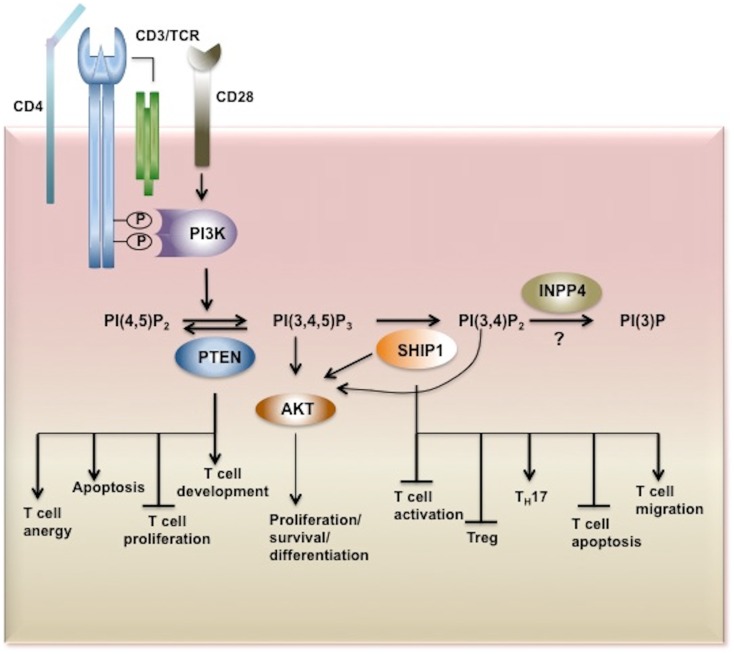
**Inositol phosphatases in T cell biology**. Upon TCR stimulation PI3K is activated and recruited to the membrane through its SH2 domain where it phosphorylates its substrate PI(4,5)P_2_ converting it to PI(3,4,5)P_3_. PI(3,4,5)P_3_ is bound by PH-domain containing proteins such as AKT, PDK1, BTK, ITK, Vav, and PLCγ triggering secondary signaling cascades and thus T cell activation, proliferation, survival, and cytokine production. PI3K signaling is tightly regulated by inositol phosphatases. PI(3,4,5)P_3_ is a substrate for three inositol phosphatases, SHIP1/2 and PTEN which hydrolyze PI(3,4,5)P_3_ to PI(3,4,)P_2_ and PI(4,5)P_2_, respectively. By limiting the cellular pool of the second messenger PI(3,4,5)P_3_, PTEN and SHIP play important functions in T cell development, proliferation, and activation. The SHIP1 product PI(3,4)P_2_ which can also recruit and activate AKT is dephosphorylated by INPP4. However the role of these 4-phosphatases in T cell biology has yet to be determined. AKT, Protein Kinase B; PDK1, phosphoinositide-dependent kinase-1, PLCγ phospholipase Cγ; ITK, IL-2-inducible T cell kinase) and BTK, Bruton agammaglobulinemia tyrosine kinase.

Inositol poly-phosphate 4-phosphatases are a class of enzymes that has two isoforms INPP4A and INPP4B, that selectively remove the phosphate group at position 4 on the inositol ring to convert PI(3,4)P_2_ to PI(3)P (112). In contrast to INPP4A, which is predominantly expressed in brain, INPP4B is highly expressed in skeletal muscle, heart, brain, and pancreas, epithelial cells of the breast, and prostate glands (113). INPP4A has been shown to regulate neuroexcitatory cell death whereas INPP4B has emerged as potent tumor suppressor in breast cancer (114, 115). The function of the INPP4A and INPP4B phosphatases in immune cells has not been investigated, although a prominent role for INPP4B in myeloid-derived osteoclast function and bone remodeling has been shown (116). Thus, further investigation of these 4′-phosphatases appears merited and particularly in cell types and immune contexts where SHIP1 has a positive signaling role (e.g., T cell survival the gut). Function of inositol phosphatases in T cells is summarized is Figure 2.

## Concluding Remarks

Consistent with studies implicating class I PI3K in T cell biology, the inositol phosphatases, SHIP1 and PTEN, have been documented to be important regulators of PI3K signaling pathway in T cells. Although SHIP1 and PTEN by dephosphorylating the PI(3,4,5)P_3_ negatively regulate PI3K signaling, their *in vivo* functions in this signaling pathway, as reveled by genetic analysis, diverge significantly. SHIP1 appears to be required for the survival of T cells *in vivo*, and particularly in the lamina propria, while PTEN inhibits T cell proliferation and prevents from lymphoproliferative syndromes. Therefore these phosphatases at the cellular level provide a fine balance of PI3K signaling necessary for the proper activation and development of T cells in order to avoid immunopathology. As SHIP1 deficiency has been shown to promote T cell apoptosis there is a significant potential for SHIP1 inhibitors, which have already shown promising results in cancer (43, 73), to be used to target autoreactive T cells in IBD conditions. Although PTEN has been shown to regulate CD4 T cell function and tolerance little is known about its role in other T cell subtypes. Further studies are therefore required to dissect PTEN signaling in T cells before therapeutic application of PTEN inhibitors in immunotherapy for cancer could be considered. In addition, the role of other lipid phosphatases SHIP2 and INNP4, which regulate the cellular pools of PtdIns (3,4,5)P_3_ and PI(3,4)P_2_, respectively, merit examination *in vivo* in the coming years using sophisticated genetic models that enable conditional and/or inducible ablation of their expression in specific T cell populations.

## Conflict of Interest Statement

William Garrow Kerr has patents pending and issues concerning the modulation and detection of SHIP activity in disease. The other authors have no conflicts to disclose.
